# Screening for Alzheimer's disease using prefrontal resting-state functional near-infrared spectroscopy

**DOI:** 10.3389/fnhum.2022.1061668

**Published:** 2022-11-28

**Authors:** Hasan Onur Keles, Ece Zeynep Karakulak, Lutfu Hanoglu, Ahmet Omurtag

**Affiliations:** ^1^Department of Biomedical Engineering, Ankara University, Ankara, Turkey; ^2^Electroneurophysiology, Istanbul Sisli Vocational School, Istanbul, Turkey; ^3^Department of Neurology, School of Medicine, Istanbul Medipol University, Istanbul, Turkey; ^4^Department of Engineering, Nottingham Trent University, Nottingham, United Kingdom

**Keywords:** fNIRS, cognitive, Alzheimer's disease, machine learning, early diagnosis

## Abstract

**Introduction:**

Alzheimer's disease (AD) is neurodegenerative dementia that causes neurovascular dysfunction and cognitive impairment. Currently, 50 million people live with dementia worldwide, and there are nearly 10 million new cases every year. There is a need for relatively less costly and more objective methods of screening and early diagnosis.

**Methods:**

Functional near-infrared spectroscopy (fNIRS) systems are a promising solution for the early Detection of AD. For a practical clinically relevant system, a smaller number of optimally placed channels are clearly preferable. In this study, we investigated the number and locations of the best-performing fNIRS channels measuring prefrontal cortex activations. Twenty-one subjects diagnosed with AD and eighteen healthy controls were recruited for the study.

**Results:**

We have shown that resting-state fNIRS recordings from a small number of prefrontal locations provide a promising methodology for detecting AD and monitoring its progression. A high-density continuous-wave fNIRS system was first used to verify the relatively lower hemodynamic activity in the prefrontal cortical areas observed in patients with AD. By using the episode averaged standard deviation of the oxyhemoglobin concentration changes as features that were fed into a Support Vector Machine; we then showed that the accuracy of subsets of optical channels in predicting the presence and severity of AD was significantly above chance. The results suggest that AD can be detected with a 0.76 sensitivity score and a 0.68 specificity score while the severity of AD could be detected with a 0.75 sensitivity score and a 0.72 specificity score with ≤5 channels.

**Discussion:**

These scores suggest that fNIRS is a viable technology for conveniently detecting and monitoring AD as well as investigating underlying mechanisms of disease progression.

## Introduction

Alzheimer's disease (AD) is the most common cause of dementia in the elderly which impacts 50 million people worldwide (Bonilauri et al., 2020). Functional abnormalities in AD likely start long before its clinical symptoms, which primarily affect executive and visuospatial abilities. Practical fNIRS systems are a promising solution for the early detection of AD because they can make quick and affordable measurements without requiring expert operators. In addition, methods based on functional measurements do not rely on patients' ability to respond to questions or follow test instructions. Medication and other therapies administered from early stages can retard disease progression and improve patients' quality of life. Currently, the diagnosis of AD relies heavily on clinical examination and tests administered by expert clinicians. Therefore, there is a need for relatively less costly and more objective methods of screening and early diagnosis.

Functional near-infrared spectroscopy (fNIRS) measures neural activity by detecting the changes in oxy- and deoxyhemoglobin concentration in the upper layers of the cortex (Yücel et al., 2021). Each light source and detector pair placed on the scalp provides an optical channel that samples the effects of cerebral blood flow and metabolism directly below the midpoint of the source–detector pair. For a practical clinically relevant system, a smaller number of optimally placed channels are clearly preferable. In this study, we investigated the number and locations of the best-performing fNIRS channels measuring resting-state activity in the prefrontal cortex (PFC). The PFC is closely associated with the high-level abilities that decline in AD (Bu et al., 2019), and resting-state recordings are easier to obtain since some patients may not be able to perform tasks. Fortunately, the PFC is also a relatively easier target for fNIRS as there is little or no hair on the forehead to impede light coupling. In order to have a wide range of locations to choose from, we used a high-density fNIRS system with 48 channels with 3.35-cm source–detector separation distances. This was shown to be the most effective separation distance in a previous study using our device (Keles et al., 2021). Resting-state recordings were collected from 21 patients and 18 healthy controls, who also completed standard neuropsychological tests.

Most studies on the applications of fNIRS in AD have addressed tissue oxygenation (van Beek et al., 2012; Viola et al., 2013; Babiloni et al., 2014; Liu et al., 2014; Marmarelis et al., 2017; Chiarelli et al., 2021), functional connectivity (Li X. et al., 2018; Nguyen et al., 2019; Niu et al., 2019; Zeller et al., 2019), and brain function during task performance (Hock et al., 1996; Fallgatter et al., 1997; Tomioka et al., 2009; Yeung et al., 2016; Ateş et al., 2017; Nguyen et al., 2019; Niu et al., 2019; Perpetuini et al., 2019). For example, a statistically significant correlation between Mini-Mental State Examination (MMSE) scores and reduced tissue oxygenation was found, and tissue oxygenation was proposed as a prognostic marker of aMCI (Viola et al., 2013).

Recent studies have also shown the utility of resting-state optical imaging for characterizing AD-related cortical functional reorganization. In a study of patients with MCI and healthy controls, it was found that the MCI group had higher right and inter-hemispheric connectivity during the resting state, but lower left and inter-hemispheric connectivity during verbal fluency tasks (Nguyen et al., 2019). Another group has found that patients with MCI showed a decreased resting-state connectivity in the PFC (Bu et al., 2019) and patients with MCI and healthy elderly controls showed lower amplitude low-frequency oscillations (0.07–0.11 Hz) measured with fNIRS in the frontal cortex when compared with young subjects (Zeller et al., 2019). The classification of AD, MCI, and normal controls was also studied with fNIRS. In the classification study, it was shown that there were significant correlations between cognitive functions and DLPFC in these patient groups (Yang and Hong, 2021). Properties of the functional connectivity network showed significant correlations with neuropsychological test scores and derived features achieved a high three-class classification accuracy (95.0%) (Kim et al., 2021). Another study used fNIRS and deep learning to distinguish not only between healthy and Alzheimer's afflicted subjects but also subjects with asymptomatic AD and dementia due to AD. They reported an 86.8% accuracy of the CNN-LSTM network when 5-fold cross-validated (Ho et al., 2022).

We found reductions in oxygenated hemoglobin in patients with AD consistent with previous studies. To further analyze the signals, we assigned univariate priority scores to the optical channels based on their extent of association with the disease state of the participants. Then we used subsets of channels selected from the highest priority channels to predict the participants' disease state from the measured signal. In this study, we used Boston Naming Test and Verbal Memory Total scores as proxies for AD severity. The results suggest that AD can be detected with a 0.76 sensitivity score and a 0.68 specificity score while the severity of AD could be detected with a 0.75 sensitivity score and a 0.72 specificity score. These were obtained with ≤5 channels on the forehead. Our results provide evidence that fNIRS is a viable technology for accurately and conveniently detecting and monitoring AD as well as investigating the underlying mechanisms of disease progression.

## Methods

### Participants

Twenty-one subjects diagnosed with AD and eighteen healthy controls were recruited for the study. The study was conducted with patients with AD who were followed up in the Medipol University Hospital Neurology Outpatient Clinic and fulfilled the inclusion criteria. An experienced neurologist examined the patients and diagnosed them with AD according to the National Institute of Neurological and Communicative Diseases and Stroke/Alzheimer's Disease and Related Disorders Association (NINCDS-ADRDA) criteria (McKhann et al., 1984). Among the patients diagnosed with clinical AD, those who were 60 years and older, had Clinical Dementia Rating Scale (CDR) scores of 1 or 2, used acetylcholinesterase inhibitors and memantine, and were capable of leading their daily lives independently were included in the study. Exclusion criteria were a history of alcohol/substance abuse, mental illnesses including schizophrenia and delirium, and epileptic seizures, brain tumors, or trauma. Patients were examined during routine therapy, where the medical treatment was not modified during the study period. For the control group, those who were 60 years and older, had MMES scores of ~25, and no psychiatric or neurological disorder history was included in the study. The Research Ethics Board of Medipol University approved this study (10840098-604.01.01-E.1925), and it was performed in agreement with the Declaration of Helsinki. All participants signed informed consent and could withdraw from the study at any time.

### Clinical and neuropsychological assessment

All subjects underwent a clinical and neuropsychological evaluation to assess their global cognitive status using the MMSE (Folstein et al., 1975) and the following cognitive domains: attention [Color-Word Stroop Test (CSWT)] (Stroop, 1935), Benton Face Recognition Test (Benton et al., 1994), memory (Wechsler Memory Scale-Revised Form (WMS-R)) (Wechsler, 1987), Verbal Memory Processes Test (SBST), language (Boston Naming Test) (Kaplan et al., 1983), visuospatial skills (Benton Judgement of Line Orientation Test) (Benton et al., 1994), Clinical Dementia Rating Scale (CDR) (Morris, 1993), Geriatric Depression Scale (GDS) (Yesavage, 1988), and Neuropsychiatric Inventory were also used for neuropsychological evaluation.

### Experimental design

The included subjects and their companions were informed briefly about the whole procedure, research, and their rights before starting the test. They were given an informed consent form to read carefully and sign. It was made sure that they understood that they could stop and leave the research at any time they wished with a guarantee of not facing any kind of consequences. After the researchers decided the given information was understood and informed consent was obtained, the subjects were asked to sit on a chair and the fNIRS device was set on the head and optodes were calibrated while the subject was asked to sit silently and in a relaxed position. After the optimal calibration was set, any kind of devices that may cause light, sound or any other distracting stimuli were turned off. When the subject confirmed that they were ready for the experiment, they were asked to sit relaxed, silent, thinking as little as possible, with their eyes shut, and try not to sleep, and then the test was started. Following the 30 s of the beginning session to check the optodes were working properly as set before the test, the 5-min recording of the resting state was started without any warning. After 5 min, the recording signal was briefly checked again and was saved in the following 15 s and the subject was informed that the test was finished.

### Imaging and data analysis

Optical imaging data were collected using a high-density fNIRS device (NIRSIT, OBELAB, Korea) with 24-light sources at 780 and 850 nm and 32 detectors, with a sample rate of 8.138 Hz. The channels overlap with parts of the dorsolateral and ventrolateral prefrontal and the upper part of the orbitofrontal and medial PFC.

For preprocessing, the detector readings were first converted into concentration changes in oxy- and deoxyhemoglobin by using the modified Beer–Lambert law (Delpy et al., 1988). We sought to diminish signal components unrelated to brain activity by band-pass filtering in the range of 0.01–0.5 Hz. This eliminated the effects of heartbeat (~1 Hz), reduced some motion artifacts that had sharp transients, and eliminated the slow baseline drift (Naseer and Hong, 2015). The frequency range of Mayer waves, with a period of ~10 s, partly overlaps with task-evoked hemodynamic responses, and hence, we did not attempt to filter them out. However, they were not expected to influence our results, as Mayer waves likely do not correlate with cognitive processes (Vermeij et al., 2014). Next, we used windowed standard deviation to quantify the presence of motion artifacts (Scholkmann et al., 2010). We aimed to minimize motion artifacts with excursions greater than those of concurrent physiological effects. We calculated the standard deviation in nonoverlapping 10-s windows and the median absolute deviation (MAD) of the set of standard deviations for each channel. Any window whose standard deviation was >4.5-MAD values away from the median was considered an outlier and excluded from subsequent analysis. We visually inspected the signals from randomly selected time segments to confirm the validity of this scheme. In addition, we confirmed that the outliers tended to occur around the same time as increases in the accelerations measured by the headset. The method captured all severe deflections, and many outliers contained only mild fluctuations, suggesting that our threshold criterion was conservative. The details of the hardware, channel locations, and signal preprocessing parameter values methods were described in a previous study (Keles et al., 2021).

**Figure 1:**
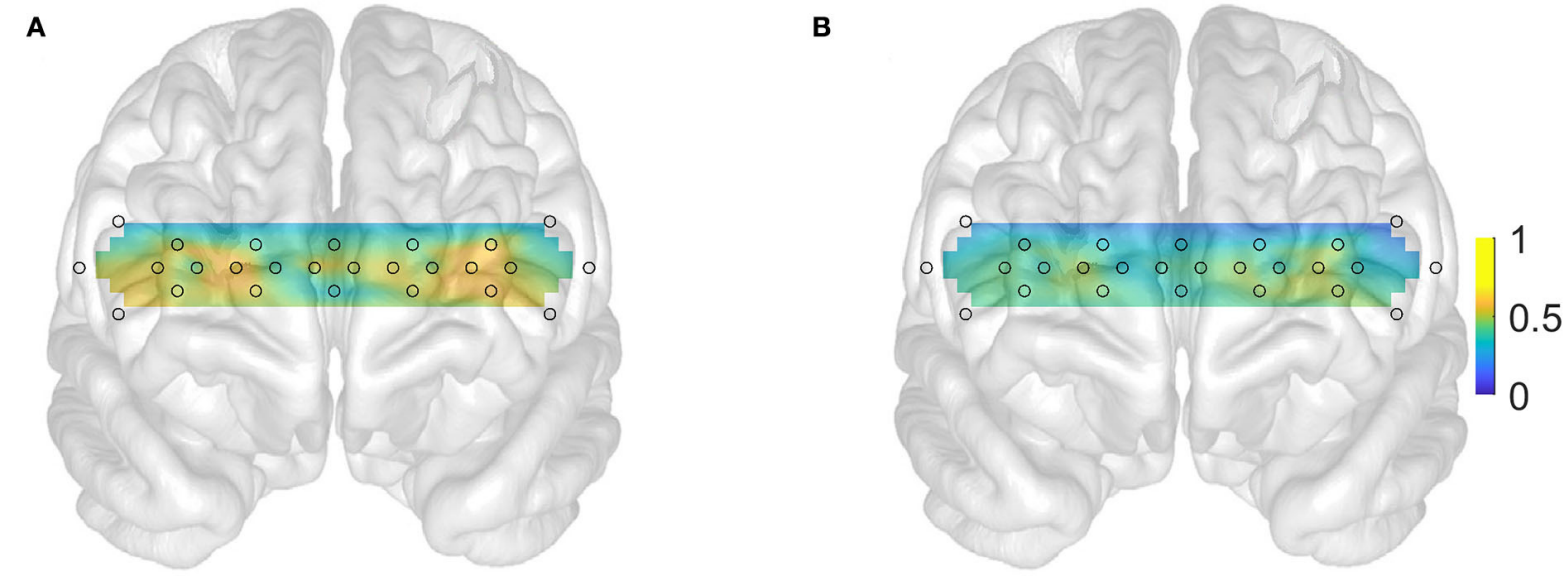
Subject averaged prefrontal cortex activations (color bar units on the right in mM) interpolated from channels with 3.35 cm separations. **(A)** Normal subjects (N = 18). **(B)** Patients (N = 21). Open gray circles indicate the location of the fNIRS channels.

In Keles et al.'s (2021) study, it was shown that the 48 channels with the longest available separation (33.5 mm) provided the best decoding ability of the subject's mental state due to their greater sampling of the cerebral tissue. Accordingly, we have used signals only from the deeper sampling channels in this study. Their topographic locations are approximately indicated by the open circles in Figure 1 and partly overlap with the bilateral orbitofrontal and ventromedial as well as the inferior regions of the dorsolateral PFC (Carlén, 2017). To determine the extent of PFC local engagement, we computed the standard deviation of the oxyhemoglobin changes in each channel over adjacent nonoverlapping 10-s windows (Tai and Chau, 2009; Holper and Wolf, 2011; Aghajani et al., 2017; Keles et al., 2021).

We have used this feature because greater hemodynamic response tends to increase the standard deviation of the signal in a window, while the mean of the signal remains close to zero. For this reason, the window mean of the signal may not be a good indicator of activation, especially when the evoked response is brief and followed by a dip. In a previous study, we explored variables such as the window mean, skewness, and kurtosis and determined that the standard deviation was the best indicator for the types of discrimination we pursued (Keles et al., 2021). Note that the standard deviation or variance has frequently been used in machine learning studies that used fNIRS signals (Tai and Chau, 2009; Holper and Wolf, 2011; Aghajani et al., 2017). Other feature extraction techniques were described by Keshmiri et al. (2018).

We repeated the calculations in this study by using only deoxyhemoglobin or by including both oxy- and deoxyhemoglobin concentration changes. However, these did not improve classifier performance relative to using oxyhemoglobin alone. In fact, deoxyhemoglobin alone resulted in slightly lower accuracies overall. Since this is a proof-of-concept investigation, we have limited this study to oxyhemoglobin concentration changes only. Henceforth, we use the term *activation* to refer to the standard deviation of the oxyhemoglobin changes.

We studied the ability of the PFC activations to discriminate between patients and healthy controls and between different subgroups of patient participants. To that end, we generated features for a machine learning approach by averaging the activation in each channel across the entire recording session of a participant. Therefore, a feature matrix contained rows consisting of individual subjects and columns consisting of channels, and its entries were the session-averaged PFC activations. The matrix contained a maximum of 48 columns (channels). Because each subject's data occurred in only one row of the feature matrix, the training and test partitions never contained data from the same subject. The corresponding binary label vector indicated (1) whether the participant was a patient or healthy control or, in a subsequent set of studies, (2) whether the patient participant obtained a high or low score on a neuropsychological test, where the high–low cut-off was taken as the median of all patients.

Using part of the data, we assessed multiple filter-type algorithms for rank ordering our features before feeding them into a classifier: filters based on (1) Pearson correlation between a feature and label vector; (2) *p*-values obtained from chi-squared tests; and (3) searching for sets of features maximally associated with the labels and minimally associated with each other. The chi-squared method was selected due to its robustness and the resulting classifier accuracies. Hence, the features were first prioritized by using chi-squared tests that determined whether each feature was independent of the label vector by calculating a *p*-value. The priority score of a feature was calculated as the natural logarithm of the reciprocal of its *p*-value. The prioritized features were then used to predict the labels of a subset of the participants using a Support Vector Machine or Linear Discriminant Analysis trained on the remaining subset of the participants. The results from the Support Vector Machine classifier were overall more accurate hence we only report them in this article. The performance of the prediction was characterized using its sensitivity and specificity defined in the following way. In the initial study, sensitivity was calculated as the ability to correctly identify a patient, and specificity was calculated as the ability to correctly identify a healthy control. In other words, considering a *positive* prediction as the prediction that the subject is a patient, the sensitivity of the method was defined as the number of true positives divided by the sum of true positives and false negatives. The specificity was the number of true negatives divided by the sum of true negatives and false positives. In the subsequent set of studies, the sensitivity was calculated as the ability to correctly identify a high-scoring patient, and specificity was calculated as the ability to correctly identify a low-scoring patient.

The accuracy was found using a 5-fold cross-validation, each repeated 20 times with different randomly selected partitions. Before proceeding with the full set of computations, we considered how the accuracy of a 3-fold, 5-fold, and 10-fold cross-validation would differ by performing a limited set of classifications to discriminate patients from normal volunteers. We considered that having too few folds may not have a sufficient number of partitions to reveal the true accuracy, while too many folds may have an insufficient number of observations per partition for proper training. We found no clear differences between these cases; however, 5-fold was selected as a potential compromise. Each cross-validation was repeated 20 times with randomly different partitions in order to generate a distribution to assess statistical significance. We selected 20 repetitions, as a greater number of repetitions did not appear to affect the result while it substantially increased the computational load. We did not use leave-one-out cross-validation as this scheme has only one possible partition and does not allow one to generate distribution and may generate biased performance estimates (Varoquaux et al., 2017). Each feature was standardized by centering and scaling with the mean and standard deviation of the corresponding column of the feature matrix. The linear kernel was selected for Support Vector Machine and its scale was computed by Matlab using a heuristic procedure. We used a fixed random number seed for the reproducibility of the results. Optimization of the box constraint and kernel scale parameters was tried to discriminate patients from normal volunteers, both based on Matlab's grid search algorithms in the range [0.001, 1000]. This significantly increased computing times without a noticeable improvement in performance; thus, in this study, we report only results based on Support Vector Machine without hyperparameter optimization.

In order to examine the ability of a small subset of the features to discriminate between the targets the above calculations were repeated by an feeding increasingly larger number of features (in order of descending priority score) into the classifier. We used the permutation method to evaluate the statistical significance of the performance indicators (Combrisson and Jerbi, 2015; Omurtag et al., 2017). In this method, the classification analysis was repeated multiple times with different randomly reshuffled label vectors which led to null distributions of sensitivity and specificities. Bonferroni-corrected *p*-values were calculated using the Wilcoxon signed-rank test that compared the null distribution with the actual distribution of sensitivity and specificities calculated from multiple different 5-fold partitions. Topographic plots of distributed activations (e.g., Figure 1) were obtained by two-dimensionally interpolating the individual values from the 48-deep sampling channels. The Matlab functions such as fscchi2, cvpartition, fitcsvm, and signrank were used in the above calculations.

## Results

**Figure 2:**
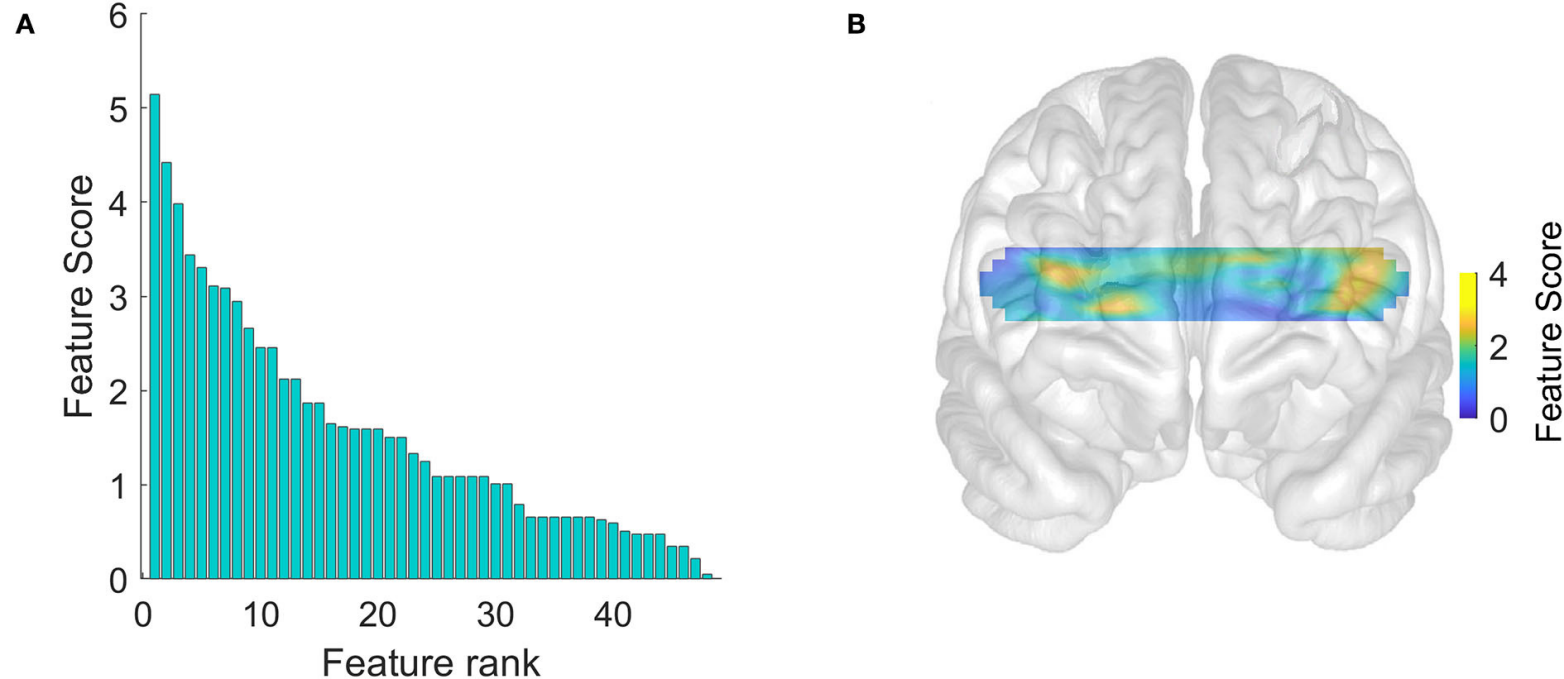
Hemodynamic feature priority scores calculated for purposes of feature selection. **(A)** Univariate feature priority scores ranked in descending order calculated using chi-squared tests. **(B)** The distribution of scores over the prefrontal cortex.

**Figure 3:**
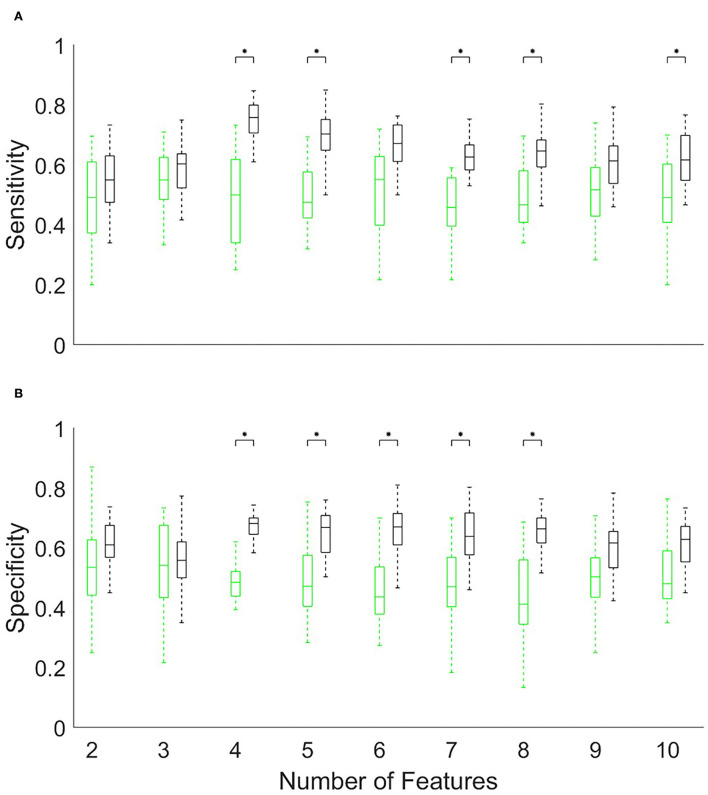
The accuracy **(A)** sensitivity and **(B)** specificity of discriminating patients (N = 21) from normal subjects (N = 18) using the Support Vector Machine and a limited number (x-axis) of the top-ranked hemodynamic features. The accuracy is found from 5-fold cross-validation repeated 20 times with different randomly selected partitions. The black boxes indicate the accuracy and the green boxes indicate the corresponding null distribution calculated by randomly permuting the labels. Statistical significance calculated from the Kolmogorov–Smirnov test is indicated using an asterisk (*p < 0.05, Bonferroni corrected). The central mark in a box indicates the median, and the bottom and top edges of the box are the 25th and 75th percentiles, while the whiskers extend to the most extreme data points not considered outliers.

**Figure 4:**
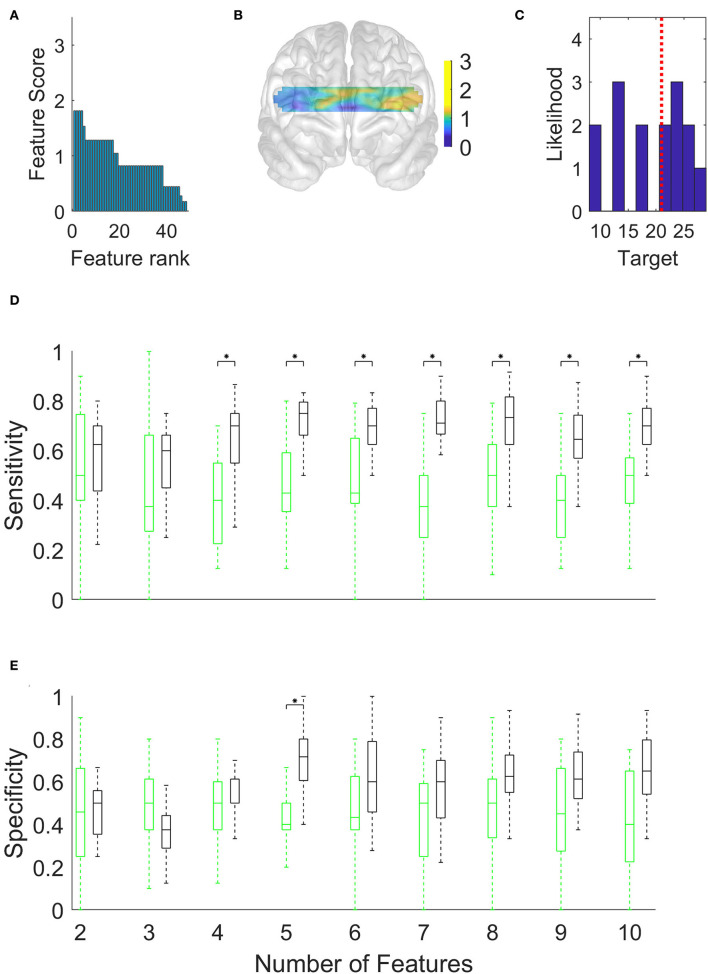
Discrimination of high-scoring patients (N = 8) from low-scoring patients (N = 7) in the Boston Naming Test, using the Support Vector Machine and a limited number (x-axis in **D** and **E**) of the top-ranked hemodynamic features. **(A)** Hemodynamic feature priority scores calculated using chi-squared tests. **(B)** Topographic distribution of feature scores. **(C)** Histogram of Boston Naming Test scores of patients. The vertical dotted red line shows the location of the median score used to distinguish high-scoring patients from low-scoring patients. **(D)** Sensitivity of discriminating high-scoring patients. **(E)** Specificity (*p < 0.05, Bonferroni corrected).

**Figure 5:**
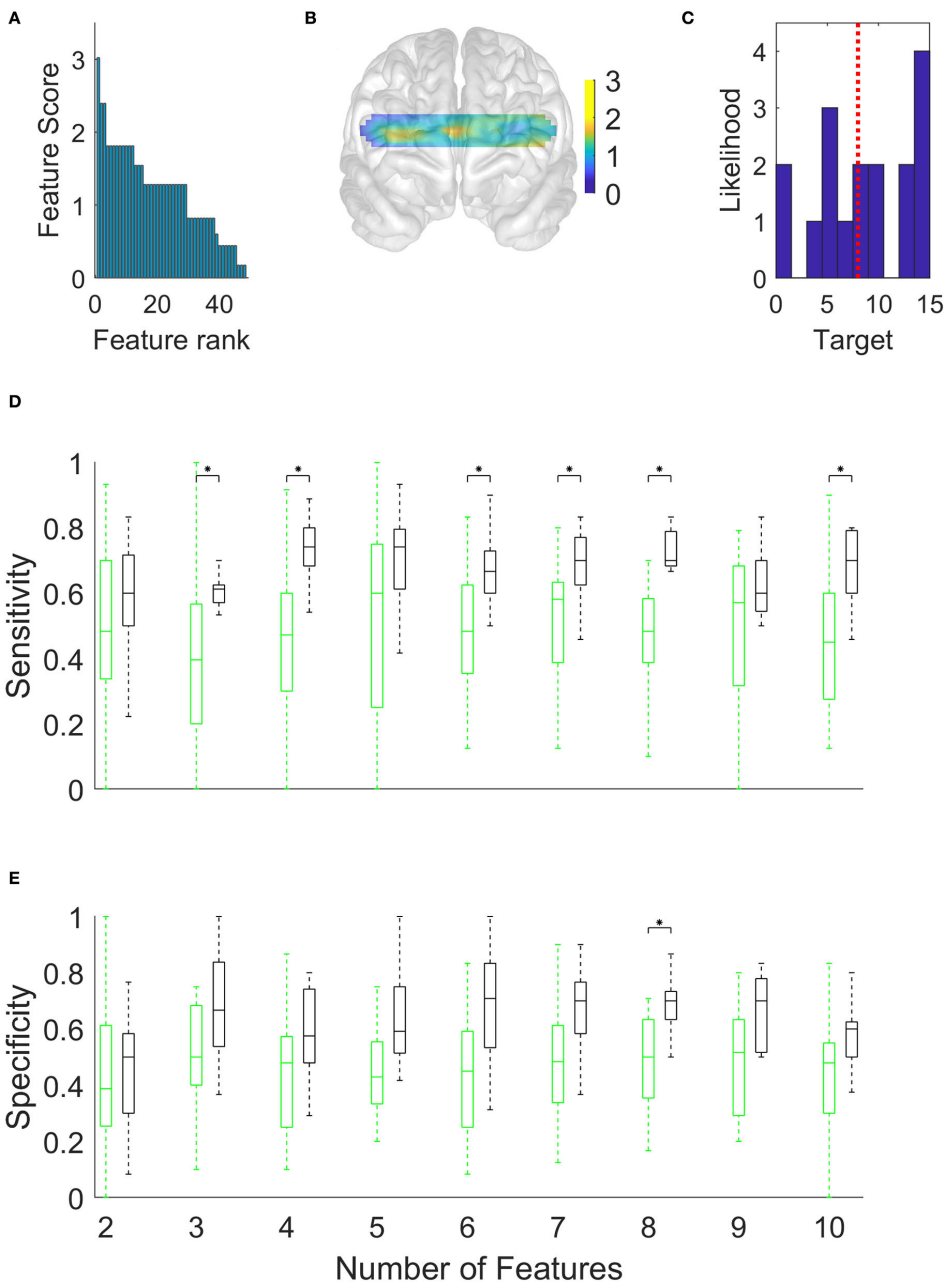
Discrimination of high-scoring patients (N = 8) from low-scoring patients (N = 9) in the Verbal Memory Total Score Recall/15, using the Support Vector Machine and a limited number of the top-ranked hemodynamic features. **(A)** Hemodynamic feature priority scores calculated using chi-squared tests. **(B)** Topographic distribution of feature scores. **(C)** Histogram of Boston Naming Test scores of patients. The dotted red line shows the median score used to distinguish high-scoring patients from low-scoring patients. **(D)** Sensitivity of discriminating high-scoring patients. **(E)** Specificity (*p < 0.05, Bonferroni corrected).

We gathered data from 21 patients with AD, 18 healthy controls. All the patients underwent a neurological and neuropsychological examination except for four patients. The results of neuropsychological tests and the demographic background of the patients with AD are shown in Table 1. In this section, we first describe and compare the prefrontal activations of patients and healthy controls (Figure 1). Then, by using the chi-squared feature priority score described in the Methods section, we investigate which optical channels were best associated with binary distinctions between subgroups of participants, such as patients vs. healthy controls (Figure 2) and high-/low-scoring patients on neuropsychological tests (Figures 4A,B,5A,B). We then use machine learning and a series of high-priority feature sets with an increasing number of features, to quantify the ability of optically imaged PFC activation to discriminate between these subgroups (Figures 3, 4D,E, 5D,E).

**Table 1:** The demographical and neuropsychological test scores of the patient group with Alzheimer's disease (AD).

	**Mean**	**S.E**.
Gender 12 F/9 M		
Age	71,76	2,25
Education	6,41	1,20
Global Evaluation MMSE Total score	20,20	1,14
Executive functions CSWT time (s)	135,42	19,82
CSWT error	13,83	2,92
Digit span (forward)	4,41	0,40
Digit span (backwards)	2,71	0,37
Category fluency	12,53	1,20
Phonemic fluency	17,87	3,44
Memory WMS-R		
Logical memory (Immediate)	4,82	0,98
Logical memory (Late)	3,76	0,94
Visual memory (Immediate)	4,20	0,89
Visual memory (Late)	1,27	0,42
Memory SBST		
Immediate item count	2,35	0,34
Total score	45,94	5,43
Recall	2,06	0,72
Total recall	8,65	1,19
Visuospatial functions BNT	19,00	1,42
BLOT	14,38	1,27
BFR	39,40	1,48
Emotional state GDS	9,33	1,45
Behavioral evaluation NPI Total score	16,87	3,19

MMSE, Mini-Mental State Evaluation; CWST, Color–Word Stroop Test; WMS-R, Wechsler Memory Scale–Revised; SBST, Verbal Memory Processes Test; BNT, Boston Naming Test; BLOT, Benton Line Orientation Test; BFR, Benton Face Recognition Test; GDS, Geriatric Depression Scale; NPI, Neuropsychiatric Inventory.

Figure 1 suggests that the subject-averaged resting-state bilateral PFC activation in patients (B) is significantly reduced relative to healthy controls (A). A comparison of Figure 1A with the known functional division of the PFC (Carlén, 2017) implicates, in particular, the dorsolateral PFC as the site of highest relative activation in the healthy controls (shown in yellow). This is further reinforced by the distribution of the feature priority scores shown in Figure 2. These suggest that the sites of activation that best discriminate the patients with AD from the healthy controls were clustered particularly (but not exclusively) in the left dorsolateral PFC. Figure 2A shows that the priority scores decreased steeply and the priority of the top three sites (located in the yellow zones in Figure 2B) was especially more salient than those of the remaining features. Figure 3 shows that the top four features achieved the highest accuracy in discriminating patients with AD from healthy controls (sensitivity 0.76 and specificity 0.68), and the accuracy tended to decline with the inclusion of an increasing number of features. The results shown in these plots are given as numerical figures in Table 2.

**Table 2:** The sensitivity and specificity of discriminating patients with AD from healthy controls.

**Number of features**	**Sensitivity** **(median ± st. dev.)**	**Specificity** **(median ± st. dev.)**
2	0.55 ± 0.10	0.61 ± 0.08
3	0.60 ± 0.09	0.56 ± 0.10
4	0.76 ± 0.08	0.68 ± 0.04
5	0.70 ± 0.09	0.67 ± 0.10
6	0.67 ± 0.08	0.67 ± 0.09
7	0.63 ± 0.06	0.64 ± 0.09
8	0.65 ± 0.08	0.66 ± 0.07
9	0.61 ± 0.09	0.62 ± 0.09
10	0.62 ± 0.09	0.63 ± 0.08

The mean and standard deviations of repeated cross-validations are shown (values are shown in the black boxes in Figure 3).

Having classified the patients with AD and the healthy controls, we turned to the more difficult task of discriminating subgroups within the patient group. We began by using the Boston Naming Test scores to allocate patients into high- and low-scoring subgroups separated by the median score (dotted red vertical line in Figure 4D). The feature priority ranking declined less steeply than before (Figure 4A), but the high-ranking features were more heavily clustered in the left dorsolateral PFC (Figure 4B). Figures 4D,E and Table 3 show that the maximum accuracy (sensitivity 0.75, specificity 0.72) could be achieved with only five features.

**Table 3:** The sensitivity and specificity of discriminating high-scoring patients from low-scoring patients in the Boston Naming Test.

**Number of features**	**Sensitivity** **(median ± st. dev.)**	**Specificity** **(median ± st. dev.)**
2	0.63 ± 0.18	0.50 ± 0.12
3	0.60 ± 0.14	0.38 ± 0.13
4	0.70 ± 0.19	0.50 ± 0.13
5	0.75 ± 0.11	0.72 ± 0.15
6	0.70 ± 0.15	0.60 ± 0.19
7	0.71 ± 0.15	0.60 ± 0.17
8	0.73 ± 0.14	0.63 ± 0.16
9	0.65 ± 0.14	0.61 ± 0.16
10	0.70 ± 0.11	0.65 ± 0.18

The median and standard deviations of repeated cross-validations are shown (values are shown in the black boxes in Figures 4D,E).

## Discussion

We have shown that resting-state fNIRS recordings from a small number of prefrontal locations provide a promising methodology for detecting AD and monitoring its progression. By using a high-density continuous-wave fNIRS system, we first verified the relatively lower hemodynamic activity in the prefrontal cortical areas observed in patients with AD (Figure 1). This is well-known from previous studies (Arai et al., 2006; Herrmann et al., 2008; Ruiz-Rabelo et al., 2015; Uemura et al., 2016; Yap et al., 2017). We, then, showed that the accuracy of subsets of optical channels in predicting the presence and severity of AD was significantly above chance (Figures 2–5). To the best of our knowledge, this is the first study in peer-reviewed literature to use machine learning to quantify the AD-related sensitivity and specificity of the resting-state fNIRS signals from the PFC.

Resting-state whole-head fNIRS data from patients with AD dementia and amnesic MCI and healthy controls were used to show that the temporal variability of functional connectivity maps was able to distinguish aMCI [area under the curve (AUC 82.5%)] or AD (AUC 86.4%) from the healthy controls (Niu et al., 2019). Further descriptions of related studies can be found in recent extensive reviews (e.g., Bonilauri et al., 2020).

Our study has focussed on discovering the locations of resting-state optical signals from the PFC that provided optimal accuracy and quantified their sensitivity and specificity. Figure 3 indicated that patients with AD and healthy controls could be discriminated with a 0.76 sensitivity score (i.e., a false-negative rate of 24% among the patient group) and a 0.68 specificity score (a false-positive rate of 32% among the healthy controls) using only four channels. Figure 4 showed similar outcomes (with higher specificity and five channels) for discriminating subgroups of patients with high or low scores in the Boston Naming Test. We have obtained similar results using the Verbal Memory Total Score Recall. Figure 4C shows that some patients in either group had scores close to the median value (the median was used to separate patients into two groups). Such close scores in different groups may have reduced the accuracy of discrimination. However, patients with scores close to the median could not be removed from this calculation since this would have reduced the already small size of the data set. Alternatively, we could have used other classification schemes [e.g., artificial neural network (ANN)] to predict the continuous range of scores, however, ANNs require a greater number of training examples than we had in our patient population.

By definition, sensitivity is reduced by the occurrence of a higher number of false negatives in the patient group, while specificity is reduced by a higher number of false positives in the healthy control group. Thus, the generally higher sensitivity observed in Figures 3, 4 indicated that PFC hemodynamics was a more robust marker among the patients than it was among healthy controls. This could be due to the greater variability of the signals in the healthy group.

The chance distribution of accuracy is shown by the green boxes in Figures 3, 4 and indicates the median and range of values obtained by repeating the 5-fold cross-validation 10 times. The repetitions, with different partitions into training/test sets and randomly reshuffled labels, yielded values that are represented by the green boxes. As expected, the chance accuracies in the Figures fluctuate around 50%. However, they remained close to 50% only if there were a sufficient number of patient responses in each of the high-/low-scoring groups (as was the case with the Boston Naming Test and Verbal Memory Total Score Recall); we used this as a criterion for excluding the other types of tests from this study.

Figures 3A,B, 4D,E suggest that the accuracy initially increased with an increasing number of optical channels (features) and then remained near a maximum or slightly declined. This was in accordance with expectations. The initial increase in accuracy was clearly due to the fact that additional features brought new information useful for discrimination. The small declines following the maximum, on the other hand, may have been due to new features adding little or no useful information but instead introducing noise into the system that obscured the differences between groups.

Our study used a technique (fNIRS) that only samples the upper layers of the cortex and may not directly reveal any pathological changes in subcortical regions. This unavoidably follows from the fact that near-infrared photons cannot reliably penetrate deeper than 2–3 cm of tissue. However, this shortcoming is mitigated by the following considerations. fNIRS provides practical and low-cost applications similar in technological footprint to electroencephalography. In addition, subcortical structures are heavily interconnected with PFC which fNIRS can investigate. In addition, fNIRS can be combined with EEG (Aghajani et al., 2017; Omurtag et al., 2017) in order to investigate neurovascular coupling (Keles et al., 2016) which has been implicated in AD-related functional changes (Babiloni et al., 2014; Liu et al., 2014). Thus, fNIRS appears to be a good choice for our study with a reasonable trade-off.

The limitations of our study and possible mitigations are as follows:

1. Only two types of tests were available with a sufficient number of patient responses. A greater number of types of neuropsychological test scores (e.g., Viola et al., 2013) would improve the validity of our findings.
2. We only collected PFC data, however, the measurement from additional areas may increase accuracy as there are differences between patients and healthy controls in parietal activation (Li R. et al., 2018). This will become more viable as better-designed headsets and optodes that can conveniently record through hair become available.
3. We only used resting-state measurements, however, data collected during cognitive or memory task performance may increase accuracy as there are clear task-evoked differences between patients and healthy controls (Arai et al., 2006; Yeung et al., 2016).

These limitations offer opportunities for further study. Our results suggest that with further improvements in instrumentation and possibly in conjunction with concurrent EEG and neuropsychological tests, a small number of fNIRS channels located in the PFC can be a valuable screening tool for diagnosing and monitoring AD.

## Data availability statement

The original contributions presented in this study are included in the article/supplementary material, further inquiries can be directed to the corresponding author.

## Ethics statement

The studies involving human participants were reviewed and approved by the Research Ethics Board of the Medipol University (10840098-604.01.01-E.1925). The patients/participants provided their written informed consent to participate in this study.

## Author contributions

Conceptualization, methodology, and writing—original draft: HK. Data curation: HK and EK. Software: AO. Supervision: LH. Validation: AO and LH. Visualization, formal analysis, and writing—review and editing: HK and AO. All authors contributed to the article and approved the submitted version.

## Conflict of interest

The authors declare that the research was conducted in the absence of any commercial or financial relationships that could be construed as a potential conflict of interest.

## Publisher's note

All claims expressed in this article are solely those of the authors and do not necessarily represent those of their affiliated organizations, or those of the publisher, the editors and the reviewers. Any product that may be evaluated in this article, or claim that may be made by its manufacturer, is not guaranteed or endorsed by the publisher.
